# Multisector Approach to Improve Healthy Eating and Physical Activity Policies and Practices in Early Care and Education Programs: The National Early Care and Education Learning Collaboratives Project, 2013–2017

**DOI:** 10.5888/pcd16.180582

**Published:** 2019-07-25

**Authors:** Teresa M. Garvin, Lisa Weissenburger-Moser Boyd, Alethea Chiappone, Casey Blaser, Mary Story, Allison Gertel-Rosenberg, Julie Shuell, Debbie Chang, Dianne Ward, Catherine Plumlee, Michael Beets, Amy L. Yaroch

**Affiliations:** 1 Gretchen Swanson Center for Nutrition, Omaha, Nebraska; 2 Duke University, Durham, North Carolina; 3 Nemours Children’s Health System, Washington, District of Columbia; 4 University of North Carolina, Chapel Hill, North Carolina; 5 University of South Carolina, Columbia, South Carolina

## Abstract

**Purpose and Objectives:**

Embedding healthy eating and physical activity best practices in early care and education settings is important for instilling healthy behaviors early in life. A collaborative partnership between Nemours Children’s Health System and the Centers for Disease Control and Prevention was created to implement the National Early Care and Education Learning Collaboratives Project (ECELC) in childcare settings in 10 states. We measured improvement at the program level by the self-reported number of best practices implemented related to healthy eating and physical activity.

**Intervention Approach:**

The ECELC implemented a collaborative model with state-level partners (eg, child care resource and referral networks) and early care and education programs. Intervention components received by program directors and lead teachers included 1) self-assessment, 2) in-person learning and training sessions, 3) action planning and implementation, 4) technical assistance, and 5) post-reassessment.

**Evaluation Methods:**

A pre–post design assessed self-reported policies and practices related to breastfeeding and infant feeding, child nutrition, infant and child physical activity, screen time, and outdoor play and learning as measured by the validated Nutrition and Physical Activity Self-Assessment for Child Care (NAP SACC) best practices instrument. The sample included 1,173 early care and education programs.

**Results:**

The number of best practices met for each of the 5 NAP SACC areas increased from pre-assessment to post-assessment approximately 6 months later and ranged from 1.5 to 4.7 best practices (*P* < .001). Almost all increases occurred regardless of participation in the Child and Adult Care Food Program, Quality Rating Improvement System, Head Start/Early Head Start, and/or accreditation status.

**Implications for Public Health:**

The innovative and collaborative partnerships led to broad implementation of healthy eating and physical activity–based practices in early care and education settings. Development, implementation, and evaluation of policy and practice-based partnerships to promote healthy eating and physical activity among children attending early care and education programs may contribute to obesity prevention in the United States.

**Summary**

**What is already known on this topic?**

Incorporating healthy eating and physical activity best practices in early care and education settings is important for instilling healthy behaviors early in life.

**What is added by this report?**

A collaborative intervention among a health care system, state-level partners, and early care and education programs increased the number of best practices being met related to breastfeeding and infant feeding, child nutrition, infant and child physical activity, screen time, and outdoor play and learning in early care and education settings.

**What are the implications for public health practice?**

Development, implementation, and evaluation of policy and practice-based partnerships to promote healthy eating and physical activity among children attending early care and education programs may help prevent obesity in the United States.

## Introduction

More than 1 in 8 children (14%) aged 2 to 5 years were obese in 2016 (1). Children who are obese are more likely to be adults who are obese and are at an increased risk for chronic diseases (ie, type 2 diabetes, cardiovascular disease, and some cancers) and premature death in adulthood (2). Furthermore, children with obesity are susceptible to depression, emotional and behavioral disorders, and poor self-esteem (3). Possibly because of comprehensive changes at the environmental and policy levels and targeted practice interventions, slight declines in obesity among children aged 2 to 5 years have been reported in some communities in the United States (1,4). These reports provide early and promising evidence for policy and practice obesity prevention efforts for this age group (1,4).

Promotion of healthy eating and physical activity (HEPA) behaviors in early care and education (ECE) settings can reduce the risk of obesity among the nearly 13 million children aged 5 years or younger who spend some portion of their week in this setting (5,6). Given the high level of exposure young children have to policies and practices in ECE programs, they are a key setting to implement strategies to improve policies and practices and contribute concurrently with other childhood obesity prevention efforts in the United States (7). Further, HEPA-based interventions targeting ECE environments, practices, and policies have demonstrated success in improving the quality of care provided (5,8–12). Preliminary evidence suggests that ECE environmental-level strategies, such as improving policies and practices related to eating, physical activity, and sedentary behaviors, may improve health behaviors of children enrolled in these programs (13–15). Although ECE provider-level interventions have demonstrated success, integrating the promotion of HEPA-based practices and policies into existing ECE systems may contribute concurrently with other initiatives aimed at childhood obesity prevention among children aged 5 years or younger.

Nemours Children’s Health System (Nemours) collaborated with the Centers for Disease Control and Prevention (CDC) to implement the National Early Care and Education Learning Collaboratives (ECELC) Project in 10 states. In 2007, Nemours developed and implemented an intervention in Delaware to promote HEPA among children in various settings, including ECE settings. A key part of the initiative was the establishment of learning collaboratives using a “train-the-trainer” model with ECE programs to identify and implement healthier policies and practices (16). The Nutrition and Physical Activity Self-Assessment for Child Care (NAP SACC) instrument (17,18) documented that all 28 ECE programs reported significant improvement in either healthy eating practices or physical activity practices, and 81% of the programs improved in both (16). In 2012, Nemours adapted this model for spread and scale, ultimately reaching 10 states (Alabama, Arizona, California, Florida, Indiana, Kansas, Kentucky, Missouri, New Jersey, and Virginia) in collaboration with CDC (19). The resulting ECELC aimed to promote healthy environments, policies, and practices related to breastfeeding and infant feeding, child nutrition, infant and child physical activity, screen time, and outdoor play and learning in ECE programs. To our knowledge, this is the largest effort to improve HEPA policies and practices in ECE programs across multiple states.

## Purpose and Objectives

**Figure 1:**
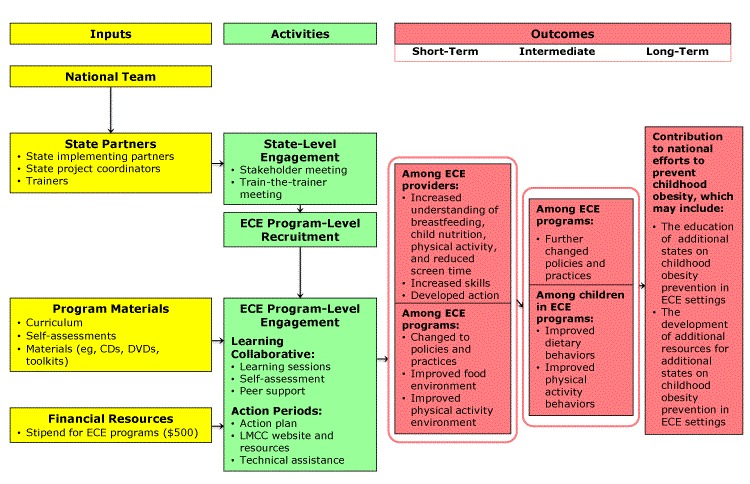
Abbreviated National Early Care and Education Learning Collaboratives Project theory of change. Abbreviations: CD, compact disc; DVD, digital versatile disc; ECE, early care and education; LMCC, Let’s Move! Child Care.

The ECELC recently ended its sixth and final year of implementation. The project established and implemented learning communities with teams of ECE providers to promote peer learning and to support and improve their HEPA policies and practices. The ECELC’s learning collaborative design is an adaptation of the Institute for Healthcare Improvement’s Breakthrough Series model (20). The ECELC was guided by a theory of change (Figure 1), which was previously applied to ECE programs (21), to outline the inputs, activities, and outcomes anticipated as part of the intervention. Evaluation efforts explored the degree to which several short-term outcomes were achieved. The primary outcome assessed throughout the evaluation was related to changes to HEPA policies and practices in ECE settings, and data were derived by using the NAP SACC instrument from 2013 to 2017 (the first 5 years of the ECELC). The purpose of this evaluation was to determine if scores from the NAP SACC instrument improved from pre-assessment to post-assessment and how similar or different these scores were across programs with regard to auxiliary federal, state, or independent agency program participation.

## Intervention Approach

**Inputs.** Inputs included the national team (ie, Nemours, CDC, and the Gretchen Swanson Center for Nutrition), state partners, program materials, and financial resources. State partners included a state implementing partner (statewide organization serving as the implementer), a state project coordinator (a staff member employed by the state implementing partner), and trainers to facilitate learning sessions and provide technical assistance. Program materials included the ECELC curriculum and training materials (eg, toolkits), as well as self-assessments (eg, NAP SACC instrument). Lastly, each ECE program received a $500 stipend to support staff time and purchasing of equipment.

**Activities.** Although Nemours provided guidance and direction on implementation, the state implementing partner had flexibility for the purposes of ownership and buy-in. It was anticipated that ECE program involvement in other state-level programs and initiatives had potential for impact on the effectiveness of the ECELC, so the model was intended to be tailorable at the state, local, and ECE program levels. ECE programs were recruited by state project coordinators through various informal methods, including personal telephone calls, online recruitment, and connections with groups such as Head Start/Early Head Start. A strength of this recruitment method was many state implementing partner agencies had existing relationships with programs and were providing them with support in other, nonhealth areas of program improvement. To be eligible to participate, ECE programs initially had to be operating in a center-based physical facility and designate a team of up to 3 people (eg, owner or director, teacher, cook) to attend 5 in-person learning sessions. During the first 5 years of the ECELC, 2,266 ECE programs were enrolled and 1,910 completed the intervention (84%).

**ECE program engagement.** The ECELC implementation cycle spanned approximately 10 months and consisted of 5 main strategies: 1) self-assessment; 2) in-person peer learning sessions, 3) action planning and implementation, 4) technical assistance, and 5) re-assessment.

**In-person peer learning.** Leadership teams and other staff participated in 5 approximately 6-hour in-person learning sessions led by trainers over a 10-month period. These sessions included didactic presentations on HEPA-based content, interactive activities, and peer sharing and support. Of the 572 programs enrolled in the first year of this project, the average number of learning sessions attended per program was 3.4 sessions (attendance data were not analyzed in later years).

**Figure 2:**
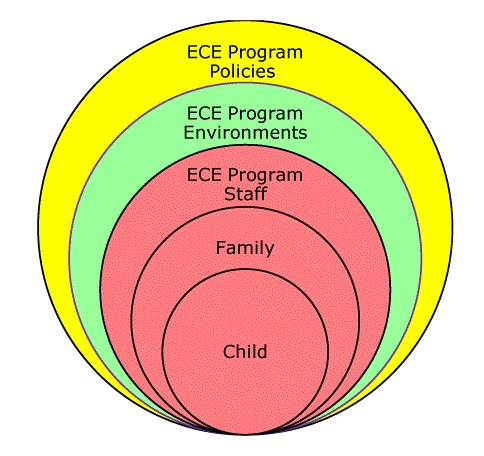
Abbreviated National Early Care and Education Learning Collaboratives Project social ecological approach.

**Action planning.** After each of the first 4 learning sessions, teams were tasked to return to their ECE programs and share what they learned. This type of peer sharing aimed to help build program-wide staff support for implementing best practices across the 5 topic areas. Each program created improvement goals with corresponding objectives based on their self-determined need (using what they learned from their self-assessment as a guide), interest, and capacity. Programs were not required to set goals for each of the 5 topic areas. Using a social ecological approach (22), programs then set action steps for each objective across 5 levels: child, family, program staff, program environments, and program policies (Figure 2).

**Technical assistance.** Individualized technical assistance at various levels of intensity, type, and frequency occurred in between learning sessions to support programs during their action planning phases. Each trainer provided technical assistance via in-person, telephone, or electronically to about 15 programs at a time. Trainers completed forms for each technical assistance interaction to describe how the technical assistance was delivered (eg, which programs received it, how much time it took, the mode of technical assistance), what NAP SACC topic area the technical assistance was related to, and if the technical assistance was related to the program’s action plan.

## Evaluation Methods

A pre–post design assessed the self-reported changes in policies and practices related to breastfeeding and infant feeding, child nutrition, infant and child physical activity, screen time, and outdoor play and learning for programs participating in the ECELC. The primary outcome data were derived from the NAP SACC instrument (17). Other data were collected before the first learning session via electronic enrollment and assessment forms including contact information, ECE program characteristics (eg, ages of children served), and state characteristics (eg, presence of a quality rating and improvement system [QRIS]). For almost all participating ECE programs, ECELC activities ended 3 months after the last learning session, and no further intervention activities were implemented. Study activities were approved by the Nemours institutional review board.

### Self-assessment of policy and practice data

Participating ECE programs completed the NAP SACC instrument after the first learning session (pre-assessment). A previous study demonstrated that 89% of NAP SACC items showed at least moderate agreement for test-retest reliability, 100% of items showed at least moderate agreement for inter-rater reliability, and 52% of items showed at least moderate agreement for validity when tested against the Environment and Policy Assessment and Observation (κ ≥ 0.20) (18). The NAP SACC instrument consisted of 5 topic areas: breastfeeding and infant feeding (23 items), child nutrition (44 items), infant and child physical activity (22 items), outdoor play and learning (20 items), and screen time (12 items) (17). Some items were specific to age groups served (ie, infants, toddlers, or preschoolers), and the rest were global (ie, applied to all 3 age groups). Programs were stratified on the basis of which age groups they served (eg, preschoolers only, toddlers and preschoolers) and were assessed according to which NAP SACC best practice items applied to their program (as opposed to individual classrooms, if applicable). Each item had 4 response options, ranging from low compliance to full compliance. For the purpose of this assessment, when the response option representing full compliance was selected, it was considered as the best practice being met (best practice met = 1). All other responses were considered to mean the best practice was not met (best practice not met = 0). Post-assessment using the NAP SACC instrument occurred during the action period before the last learning session.

### Analysis

The inclusion criteria for this evaluation included center-based ECE programs that participated in the ECELC through June 2017. Family child care homes were excluded from this analysis because of the heterogeneity of that setting compared with center-based ECE programs. The eligible pool of ECE programs was 1,879. Programs were further excluded from analysis if they served any combination of age groups other than preschoolers only; toddlers and preschoolers; or infants, toddlers, and preschoolers, the 3 most common configurations of age groups served in this sample. To align with the self-determined, pre–post design of this evaluation, programs were further excluded from topic area-specific analyses if they did not respond to at least one item in both the pre-assessment and post-assessment for that topic area of the NAP SACC instrument.

Primary comparisons of NAP SACC instrument change scores were conducted by using a longitudinal linear mixed model where the outcome variables were the 5 NAP SACC topic area scores measured for each ECE program at pre-assessment and post-assessment. Covariates contained in every model were: wave (denoting time point), age-groups served (except for breastfeeding and infant feeding, because it was administered only to programs serving infants, toddlers, and preschoolers), implementation cycle, wave-by-age-groups-served interaction (except for breastfeeding and infant feeding), and wave-by-implementation-cycle interaction. Models for specific program characteristics (eg, participation in the Child and Adult Care Food Program [CACFP]) also included the identified characteristic and a wave-by-characteristic interaction. The interrelatedness of a program’s pre-assessment and post-assessment scores was captured by using a first-order autoregressive structure covariance pattern (1). We used *t* tests to assess overall effects of program characteristics on change scores without controlling for covariates. Significance was set at a 2-sided α level of *P* < .05.

## Results

The final analytic sample included 1,173 ECE programs (62% of eligible programs), of which 260 served preschoolers only; 229 served toddlers and preschoolers; and 684 served infants, toddlers, and preschoolers (Table 1). Almost all of the ECE programs offered full-day care (93%), approximately half operated as nonprofit organizations (47%), 19% were designated as Head Start/Early Head Start, 14% were school-based, 18% were faith-based, and 1% were military-based. Most programs participated in the CACFP (62%), 34% reported being accredited, and 39% reported participating in their state’s QRIS. Meals and snacks most frequently served were breakfast (81%), lunch (87%), and afternoon snack (90%).

**Table 1:** Characteristics of Early Childhood Education Programs (N = 1,173), Partnership to Implement the National Early Care and Education Learning Collaboratives Project in Childcare Settings in 10 US States, 2013–2017

Characteristic	No. (%)
**Combination of age groups served^a^**	
Preschoolers	260 (22.1)
Toddlers and preschoolers	229 (19.5)
Infants, toddlers, and preschoolers	684 (58.3)
**Program type**	
Nonprofit	554 (47.2)
Private	271 (23.1)
Head Start/Early Head Start	217 (18.5)
School-based	162 (13.8)
Faith-based	208 (17.7)
Military	9 (0.8)
Native American–tribal, migrant, or seasonal	5 (0.4)
**Operating hours^a^**	
Half-day care available	480 (40.9)
Full-day care available	1,086 (92.6)
24-Hour care available	20 (1.7)
Participated in Child and Adult Care Food Program	731 (62.3)
Accredited	393 (33.5)
Participated in state’s Quality Rating and Improvement System	456 (38.9)
**Food service^a^^,^ b**	
Breakfast	939 (80.6)
Morning snack	404 (34.4)
Lunch	1,018 (86.8)
Afternoon snack	1,051 (89.6)
Dinner	72 (6.1)

a Items may not total 1,173 because of nonresponse and differences in which data on characteristics were collected in each cycle.

b Forty-one programs reported that they did not serve snacks or meals.

The number of best practices met for each of the 5 NAP SACC topic areas significantly increased over the 10 months from pre-assessment to post-assessment (ranging from 1.5 to 4.7 best practices; *P* < .001) (Table 2). The percentage change was the lowest for child nutrition, where an improvement of 4.7 best practices resulted in a 20% improvement. It was the highest for outdoor play and learning, where an improvement of 2.4 best practices resulted in a 44% improvement.

**Table 2:** Overall NAP SACC Change Scores Per Topic Area (N = 1,173), Partnership to Implement the National Early Care and Education Learning Collaboratives Project in Childcare Settings in 10 US states, 2013–2017^a^

NAP SACC Topic Area	Pre-Assessment	Post-Assessment	Change Score^b^	Percentage Improvement
Breastfeeding and infant feeding	9.7	12.6	2.9	29.9
Child nutrition	23.3	28.0	4.7	20.2
Infant and child physical activity	8.0	11.3	3.3	41.3
Outdoor play and learning	5.5	7.9	2.4	43.6
Screen time	5.2	6.7	1.5	28.9

Abbreviation: NAP SACC, Nutrition and Physical Activity Self-Assessment for Child Care.

a Analysis included early childhood education programs that responded to at least one item in the corresponding section of NAP SACC at pre-assessment and at least one item in post-assessment.

b *P* < .001.

This evaluation focused on the potential for 4 program characteristics to influence NAP SACC scores: CACFP, QRIS, Head Start/Early Head Start, and accreditation (Table 3). Of these, Head Start/Early Head Start programs, those participating in CACFP, or accredited programs had significantly higher scores at pre-assessment (for all 5 topic areas) than those that did not. Participation in these supplemental initiatives was associated with pre-assessment scores being between 0.5 to 6.8 best practices higher. QRIS participation was associated with higher pre-assessment scores among 4 of the 5 topic areas at pre-assessment, with outdoor play and learning being the exception. Head Start/Early Head Start programs improved by 1.6 fewer best practices in Child Nutrition compared with ECE programs that were not Head Start/Early Head Start designated (*P* < .001). Additionally, accredited programs improved with regard to screen time, but by a smaller amount (0.4 fewer best practices; *P* = .02) when compared with nonaccredited programs.

**Table 3:** ECE Program Characteristics Associated with NAP SACC Pre-assessment and Change Scores, Partnership to Implement the National Early Care and Education Learning Collaboratives Project in Childcare Settings in 10 US states, 2013–2017

NAP SACC Topic Area/ECE Program Characteristic	Difference at Pre-Assessment				Difference in Change Score			
	No^a^	Yes^b^	Estimated Difference in Score^c^	*P* Value	No^d^	Yes^e^	Estimated Difference in Score^f^	*P* Value
**Breastfeeding and infant feeding**								
CACFP	8.52	10.41	1.96	<.001	3.07	2.68	−0.48	.20
QRIS	9.15	10.22	0.85	<.001	2.66	3.04	0.61	.19
Head Start/Early Head Start	9.66	10.65	1.56	<.001	2.76	3.14	0.50	.43
Accreditation	9.33	10.41	1.18	<.001	3.03	2.48	−0.46	.23
**Child nutrition**								
CACFP	19.76	25.32	5.57	<.001	5.03	4.59	−0.46	.25
QRIS	23.00	23.53	0.44	.04	4.77	4.68	0.01	.69
Head Start/Early Head Start	22.02	28.24	6.79	<.001	5.09	3.49	−1.64	<.001
Accreditation	22.94	23.76	1.04	.002	4.79	4.84	−0.09	.83
**Infant and child physical activity**								
CACFP	7.22	8.45	1.24	<.001	3.20	3.37	0.15	.57
QRIS	7.66	8.51	0.76	<.001	3.25	3.31	0.07	.61
Head Start/Early Head Start	7.78	9.02	2.12	<.001	3.44	2.75	−0.64	.05
Accreditation	7.64	8.65	0.92	<.001	3.20	3.54	0.38	.18
**Outdoor play and learning**								
CACFP	5.19	5.67	0.46	.03	2.53	2.53	−0.10	.73
QRIS	5.30	5.81	0.59	.13	2.56	2.15	−0.37	.23
Head Start/Early Head Start	5.43	5.79	0.48	.02	2.47	2.54	0.17	.60
Accreditation	5.11	6.13	1.00	<.001	2.63	2.07	−0.56	.05
**Screen time**								
CACFP	4.86	5.36	0.49	<.001	1.42	1.51	0.05	.77
QRIS	4.95	5.57	0.62	<.001	1.55	1.35	−0.14	.43
Head Start/Early Head Start	5.05	5.70	0.87	<.001	1.47	1.46	0.04	.85
Accreditation	4.95	5.65	0.70	<.001	1.61	1.23	−0.39	.02

Abbreviations: CACFP, Child and Adult Care Food Program; ECE, early care and education; NAP SACC, Nutrition and Physical Activity Self-Assessment for Child Care; QRIS, quality rating and improvement system.

a The arithmetic mean of pre-assessment scores for programs without the characteristic (ie, non-CACFP).

b The arithmetic mean of pre-assessment scores for programs with the characteristic (ie, CACFP).

c Model-estimated pre-assessment score difference between levels of characteristic (yes and no) after controlling for differences due to time, cycle, child age groups served, and relevant interaction effects.

d The arithmetic mean of change scores for programs without the characteristic (ie, non-CACFP).

e The arithmetic mean of change scores for programs with the characteristic (ie, CACFP).

f Model-estimated change score difference between levels of characteristic (with and without) after controlling for differences due to time, cycle, child age groups served, and relevant interaction effects.

## Implications for Public Health

We found that the ECELC was an effective multisector approach to promote important changes to policies and practices in ECE programs related to breastfeeding and infant feeding, child nutrition, infant and child physical activity, outdoor play and learning, and screen time. Findings suggest that the ECELC fulfilled a key, short-term outcome, in that ECE programs made changes to policies and practices that evidence suggests may lead to improved food and physical activity environments for young children in ECE settings (13–15). 

An evaluation conducted after the ECELC was initially implemented found that a subsample of ECE programs that had participated in the ECELC maintained improvements in NAP SACC best practices for all topic areas one year later (23), suggesting potential for the ECELC to also achieve intermediate outcomes outlined in the theory of change. On average, the percentage change was the lowest for child nutrition, where a mean increase of 4.7 best practices resulted in a 20% improvement, and highest for outdoor play and learning, where a mean increase of 2.4 best practices resulted in a 44% improvement. These improvements are proportionate to pre-assessment scores, so it is important to consider how much room ECE programs had to grow. ECE programs may have had more best practices to choose from with regard to outdoor play and learning, making it easier to improve in that area. 

A key part of the ECELC included building collaborations across ECE programs and with community partners (20). National partners represented health care (Nemours), government (CDC), and nongovernmental organization (Gretchen Swanson Center for Nutrition) sectors, who worked with state-level implementation partners (eg, child care resource and referral networks and health departments) and participating ECE programs to implement healthier practices and policies. National, state, and local partners worked collaboratively to implement the initiative, gather the data, and demonstrate the effectiveness of the ECELC, thus building and applying a strong evidence base for adopting a learning collaborative model to promote the adoption of HEPA-based practice and polices among ECE programs (24). Findings from this evaluation may inform future research, especially efforts to measure any direct effect on population health, reactions or expectations for participation and performance among sectors (eg, health care, government, nongovernmental organization, states, localities), or catalytic changes and spillover effects to inform a clearer view of how multisector partnerships contribute to population health improvement (24).

In 2017, Richter et al urged scaling up of effective interventions for early childhood development by integrating into systems of health, education, and social and child protection, expressing that health and nutrition services are ideal starting points because of the relative affordability (25). Overall, programs participating in CACFP, QRIS, or Head Start/Early Head Start, or accreditation programs reported meeting significantly more best practices at pre-assessment compared with their counterparts that were not participating per each of these programs or initiatives. This expected finding was consistent with other scientific literature (26,27) and was likely a result of the availability of resources via federal funding and educational materials and trainings, especially for Head Start/Early Head Start and CACFP programs. CACFP, Head Start/Early Head Start, QRIS, and accreditation require ECE programs to adhere to a set of quality standards related to food, physical activity, and screen time, which may have promoted best policies and practices among ECE programs before the start of the ECELC, and also could have indicated ECE programs that were more equipped or ready to change. It is probable that compared with QRIS or accreditation, CACFP and Head Start/Early Head Start focus more on children’s access to healthy environments. 

Although these findings are unsurprising, they help illustrate how the ECELC can operate synergistically among other HEPA-based efforts among ECE settings. Conversely, differences in change scores by program characteristics were reported only in the topic areas of child nutrition and screen time for Head Start/Early Head Start and accredited programs, respectively, and participation in these external initiatives for these topic areas was associated with lesser improvement. When programs did not improve at the same rate, it may have been because they were already meeting more best practices at the start of the ECELC. Regardless, an opportunity exists to explore why seemingly high-quality programs voluntarily enroll in HEPA-based interventions, as well as how to reach ECE programs in greater need for improvement. That programs usually improved at the same rate whether they participated in external initiatives or not suggests that the ECELC may help fill a gap in resources, educational materials, and/or setting standards among all ECE programs. 

The multisector learning collaborative model also helped facilitate ECE programs to be more ready to meet standards, a need that exists among CACFP programs (28,29). A next step for the multisector collaborative model may be to partner with leaders of external initiatives (eg, US Department of Agriculture, Head Start/Early Head Start, accreditation agencies) to incorporate elements of the learning collaborative model into existing frameworks. Overall, the lack of differences in change from pre-assessment to post-assessment across ECE program characteristics in combination with the improvement in best practices met in the overall sample suggests that the multisector partnership may be complementary and not duplicative to outside support. It also demonstrates that the learning collaborative model, including tailorable components like technical assistance (30), may be generalizable to both well-resourced and poorly-resourced ECE programs. More specifically, the learning collaborative model can tailor training approaches toward guiding poorly-resourced ECE programs from partial compliance of best practices (as identified on their NAP SACC) to full compliance (30). As for well-resourced ECE programs, technical assistance can be allocated toward providing program-specific feedback rather than providing nonprogram-specific resources or guidance.

Although currently no federal nutrition or physical activity standards are enforced in ECE programs and most states lack meaningful regulations related to HEPA (31), state-level promotion of HEPA in ECE may support best practices. At the same time, further development is needed across most states (32,33). For example, ECE programs in Missouri may have been exposed to the Missouri Eat Smart and MOve Smart Guidelines for Child Care, which recommends ECE programs meet rigorous dietary and physical activity standards above the minimum requirements (34). Considering ways to enforce HEPA standards in ECE programs through a state’s QRIS, such as Arizona’s Quality First system, may also encourage and promote meeting best practices. Although they were not asked about specific accrediting agencies, accredited programs in this study may have also been encouraged or incented to meet best practices. A study of 185 licensed, full-time ECE programs that were assessed about program structure, staff training and behavior, and sedentary environment also showed that accreditation through the National Association for the Education of Young Children was associated with more physical activity–promoting practices (35). Success in the ECELC underscores how implementing standards in ECE settings can be critical and ultimately contribute to reduced risk for obesity among children younger than 5 years.

The US Departments of Health and Human Services and the US Department of Education have stated that ECE programs and providers must receive proper education and training, as well as fair compensation, to promote high-quality experiences for all children in these settings (36). However, in general, the ECE system in the United States lacks resources and funding to support adequate training and compensation for early childhood educators across all settings, making it difficult to support and promote best practices (36). Furthermore, ECE providers are among the lowest-paid workers in the United States and often do not receive health insurance or retirement benefits (37). Although the ECELC was an effective multisector model to promote important changes to HEPA policies and practices in ECE programs, integrating obesity prevention programming in a way that creates additional workload without augmented compensation could potentially lead to challenges, such as resistance or increased employee turnover. A 2014 study conducted in Ohio determined that financial stability was crucial to quality in ECE settings, and that most programs that were considered high-quality had supplemental revenue streams (38). Authors suggested that states could support ECE by subsidizing programs to ensure that quality care is available for working families (38). Because state-level systems (eg, QRIS, health promotion initiatives) affect ECE programs, it may be feasible to explore necessary supports for integrating learning collaboratives into statewide efforts. This may involve estimating resources required (eg, human, technical, financial), considering who might perform key functions at the state-level to reduce overlap, ensure consistent evaluation over time, and determine feasibility and associated cost of these systems (39). In this study, state implementing partner agencies had existing relationships with ECE programs and were already providing them with support in other, nonhealth areas of program improvement. State implementing partner agencies have the opportunity to identify and build on local strengths and also focus on areas of greatest need, which may contribute to more effective HEPA promotion in a learning collaborative setting. Training and compensating ECE providers is a key aspect of the US labor force having access to quality child care, so another federal agency that may have a stake in supporting ECE providers would be the US Department of Labor.

These findings should be interpreted with caution because of the contextual differences across locations as well as measurement-based limitations. Although a strength of this intervention is that strategies were consistent throughout the 6 years of the intervention, the degree to which ECE programs received intervention components (ie, technical assistance) varied. In addition, the intervention was supported via funding (eg, ECE programs were incentivized by $500) and staff support. Reasons for programs enrolling or dropping out of the intervention were not tracked consistently, and participating programs may have been motivated to change. Despite these limitations, results from annual evaluations were used to inform the development and improvement of the ECELC, contributing to the spread and scaling of the intervention across multiple states with consistent results (40). The reproducibility of results provides evidence that this model allowed, as intended, for tailoring at the state, local, and ECE program levels, which may be a key component for ensuring sustained reach of the learning collaborative model. Although a more robust, less subjective measure would have been appropriate to assess intervention impact, NAP SACC is a stable and reasonably accurate instrument for use with child care interventions (18) and has been used widely in ECE settings. Because the NAP SACC pre-assessments occurred after the first learning session and the post-assessment occurred before the last learning session, “true” pre–post data were not collected. We were unable to use a control group and did not have the resources to fully explore and delineate other factors beyond the ECELC (eg, other initiatives or campaigns) that also may have contributed to the positive changes. Furthermore, we did not explore changes in behaviors at the child level, so we cannot comment on whether the ECELC reduced risk for obesity among young children, although expert opinion is that HEPA-promoting environments have the potential to influence individual behaviors (22). Last, this evaluation did not include a cost analysis, although the development of cost-effective adaptations to the delivery of the ECELC may help facilitate the adoption, support, and sustainability of the model in additional states, communities, and ECE programs.

This evaluation demonstrated that the innovative and collaborative partnership led to broad implementation of best practices related to breastfeeding and infant feeding, child nutrition, infant and child physical activity, outdoor play and learning, and screen time in ECE settings. The ECELC model was found to be complementary and not duplicative to existing programs and initiatives (eg, CACFP). Findings also suggest that the multisector learning collaborative model may be generalizable to both well-resourced and poorly resourced ECE programs and that an opportunity exists to engage additional sectors (ie, federal departments, state and local governments, state-level QRIS systems, and additional ECE programs) to collaborate and work toward shared goals, such as developing a healthy workforce through fostering early development. By implementing policies and practices in these settings, there is potential for reaching approximately 1 in 4 children aged 5 years or younger and their families. Development, implementation, and evaluation of policy and practice-based partnerships to promote HEPA among children attending ECE programs may contribute to obesity-prevention in the United States.
